# Mental health in patients affected by atopic dermatitis: which effects of treatment with dupilumab?

**DOI:** 10.1097/YIC.0000000000000511

**Published:** 2023-12-22

**Authors:** Silvia Mariel Ferrucci, Simona Tavecchio, Gregorio Nicolini, Luisa Angileri, Alessandro Ceresa, Giulia Del Tordello, Emilio Berti, Angelo Valerio Marzano, Massimiliano Buoli

**Affiliations:** a Dermatology Section, Fondazione IRCCS Ca’ Granda, Ospedale Maggiore Policlinico; b Department of Pathophysiology and Transplantation, University of Milan; c Department of Mental Health, Department of Biomedical and Clinical Sciences Luigi Sacco, Luigi Sacco Hospital, University of Milan; d Department of Neurosciences and Mental Health, Fondazione IRCCS Ca’ Granda Ospedale Maggiore Policlinico, Milan, Italy

**Keywords:** anxiety, atopic dermatitis, depression, dupilumab, internet addiction

## Abstract

Atopic dermatitis (AD) is an inflammatory skin disease. Patients with AD are prone to develop anxiety and mood disorders. Aim of this study is to investigate if treatment with dupilumab may improve mental health status of patients affected by AD.

A total of 66 patients with severe AD were included: 24 subjects were candidate or have just started (one month) treatment with dupilumab, and 42 have been in treatment for one year.

25.8%, 30.3%, and 45.5% of the total sample showed, respectively, clinically significant anxiety, depression, and symptoms of Internet addiction. Patients with anxiety symptoms resulted to have more severe AD, more sleep problems (*P* = 0.028), less quality of life (*P* = 0.001), more severe depressive symptoms (*P* < 0.001), to be more frequently women (*P* = 0.016), to be less frequently treated with dupilumab for one year (*P* = 0.025). Similarly, patients with clinically significant depressive symptoms resulted to have more severe AD, more sleep problems (*P* = 0.003), less quality of life (*P* < 0.001), more severe anxiety symptoms (*P* < 0.001), to be less frequently treated with dupilumab for one year (*P* = 0.008).

Patients with AD treated for one year with dupilumab showed a better mental health profile in terms of less severe anxiety and depression with respect to their counterparts.

## Introduction

Atopic dermatitis (AD) is a prevalent and disabling condition affecting until 7% of general adult population (Eckert *et al.*, 2019). Different factors contribute to the severity of this condition including genetic predisposition (Nedoszytko *et al.*, 2020), T-cell-driven over-inflammation (Langan *et al.*, 2020) and environmental factors such as air pollution (Lopez *et al.*, 2021) or type of diet (Rustad *et al.*, 2022). The course of this condition can be complicated by different medical comorbidities including asthma or allergic rhinitis (Langan *et al.*, 2020). In the last years, different authors have observed an increased risk of psychiatric conditions in patients affected by AD, especially depressive and anxiety disorders (Shin *et al.*, 2016; Schonmann *et al.*, 2020). Different reasons were advocated to explain the frequent comorbidity between AD and affective disorders including shared underlying biological dysfunctions (e.g. immune system over-reactivity), worries for skin lesions (Ferrucci *et al.*, 2021) and psychological distress associated with dermatological disease (Namli *et al.*, 2022). Specifically, Namli and co-authors (Namli *et al.*, 2022) highlighted that patients with a longer duration of dermatological illness are more vulnerable to develop stress disorders. In addition, nocturnal itch may compromise the quality of sleep thus triggering the development of depression in biologically predisposed subjects (Bawany *et al.*, 2021). Similarly, patients with severe manifestations of AD may recur to self-medication with substances of abuse to have relief from insomnia and concerns about their health (Fan *et al.*, 2022). On the other hand, subjects with AD may spend a lot of time on internet to look for information about their dermatological condition and possible interventions (Pilz *et al.*, 2022).

Dupilumab, a fully human mAb inhibiting signaling of both interleukin IL-4 and IL-13, is labeled for the treatment of moderate-severe AD in adults and adolescents (Ferrucci *et al.*, 2022; Stingeni *et al.*, 2022). Registration trials demonstrated that this compound not only ameliorated the dermatological disease but also improved quality of life of patients affected by AD (Simpson *et al.*, 2016). On the other hand, given the role of over-inflammation in the pathogenesis of psychiatric disorders, in the last years different research groups have evaluated the effectiveness of immune modulators in patients affected by autoimmune conditions (psoriasis and Crohn’s disease) and clinically relevant depressive symptoms (Vasiliu, 2022). In this sense, treatment with dupilumab should improve affective symptoms in patients affected by AD by 3 mechanisms: (1) mitigating the pro-inflammatory status typical of patients with AD and psychiatric conditions (Caldiroli *et al.*, 2022); (2) improving the nocturnal itch associated with sleep disorders that can exacerbate mood disorders (Buoli *et al.*, 2018); (3) ameliorating dermatological disease that contributes to relevant psychological distress especially in subjects with long duration of illness (Namli *et al.*, 2022).

In the light of these considerations, the objectives of this study are to evaluate the presence of psychiatric comorbidities in patients with AD and, possibly, to verify whether therapy with the mAb dupilumab is effective also on these aspects.

## Methods

In this study, we recruited a sample of 66 patients suffering from severe AD: 24 subjects were candidates or had just started (1 month) treatment with dupilumab, while 42 patients had already been treated with this compound for 1 year. The presence of medical comorbidity or a psychiatric diagnosis did not represent reason of exclusion from the study.

The subjects were selected from those followed up at Dermatology Outpatient Clinic, Fondazione IRCSS Ca’Granda Ospedale Maggiore Policlinico in Milan in the months between October 2021 and March 2022. During the visits clinical information were collected and rating scales were administered to the patients. Data about the following variables were obtained: age, gender, serum total IgE (kU/l).

The severity of AD and mental health of these patients was assessed by the following scales: Patient Oriented Eczema Measure (POEM), the Itch Numerical Rating Scale (itch-INRS), Physician Global Assessment (PGA), and Eczema Area and Severity Index (EASI), Dermatology Life Quality Index (DLQI), Hospital Anxiety and Depression Scale (HADS), Sleep Quality Numeric Rating Scale (SQ-NRS), Internet Addiction Test (IAT), Drug Abuse Screening (DAST) and lie/bet questionnaire. Some of these scales were administered to the patient by the clinicians, while others were completed by the patient himself. Some of these scales are supported by validation studies conducted in Italian clinics: the itch-INRS (Storck *et al.*, 2021), DLQI (Mazzotti *et al.*, 2005), HADS (Costantini *et al.*, 1999), IAT (Faraci *et al.*, 2013)

POEM evaluates AD severity from patients’ perspective with a score ranging from 0 to 28 (Charman *et al.*, 2013). The itch-INRS is a simple 1-item self-rated scale used to assess the severity of this single symptom (score from 0 to 10: the latter corresponding to the worst itch) (Phan *et al.*, 2012). The PGA measures AD global severity by a 6-point scale (0: clear, 1–2: mild, 3: moderate, 4–5: severe) (Gooderham *et al.*, 2018). The EASI is a tool to assess the severity and extension of AD: the score of severity of AD is obtained considering the redness, thickness, scratching and lichenification of body regions (head and neck, trunk, upper limbs and lower limbs) (Hanifin *et al.*, 2001). The DLQI is a pioneering instrument to assess the quality of life of patients affected by dermatological diseases and it consists of 10 items (score 0–3) with a possible total score from 0 to 30, this latter score corresponding to the maximum negative impact of the skin disorder on patients’ quality of life. A score ≥ 6 indicates at least a moderate negative effect of illness on quality of life (Birdi *et al.*, 2020). The HADS is usually administered in patients with medical conditions to evaluate the presence and severity of affective symptoms: It consists of 2 subscales (one for depression and one for anxiety) of 7 items (Stubbs *et al.*, 2022). A score more or equal to 8 in each subscale of HADS indicates the presence of clinically relevant anxiety or depression (Stubbs *et al.*, 2022). The SQ-NRS is a single-item tool to measure the quality of sleep in the last 24 h with a score ranging from 0 (the best quality of sleep) to 10 (the worst quality of sleep) (Martin *et al.*, 2009). IAT measures the presence and severity of internet addiction (IA) and consists of 20 statements rated on a 5-point scale ranging from 0 to 5 (Young, 1998). A score ≥ 30 reveals a clinically significant IA (Young, 1998). The DAST is a 28-item self-report screening instrument for the abuse of drugs other than alcohol (Skinner, 1982). A score of 0 or 1 can be attributed to each item: a total score between 6 and 11 indicates a possible substance use disorders, a total score ≥ 12 is suggestive of a substance use problem (Skinner, 1982). The Lie/Bet Questionnaire is a two question screening tool for the identification of subjects with potential pathological gambling. A ‘Yes’ response to one or both items indicates that further assessment is needed to verify the presence of pathological gambling (Johnson *et al.*, 1997).

Descriptive analyses of the total sample were performed. The groups identified by clinically significant scores for anxiety, depression, and IA, respectively, were compared using independent-sample t-tests for continuous variables and χ^2^-tests for qualitative ones (with calculation of odds ratios where applicable). Statistical significance was set at *P* ≤ 0.05. Finally, rating scale scores (as well as age, gender distribution and serum total IgE) were compared between the two groups of treatment (candidates/recent introduction of dupilumab versus 1 year of dupilumab) by independent-sample t-tests. Benjamini-Hochberg false discovery rate correction was applied for multiple comparisons (Green and Diggle, 2007). Statistical significance was set at *P* ≤ 0.05.

## Results

Descriptive analyses of the total sample are summarized in Table 1. Respectively, 25.8%, 30.3%, and 45.5% of the total sample showed clinically significant anxiety, depression, and IA symptoms. Two patients in the group of subjects treated with dupilumab for 1 year had a psychiatric diagnosis and were followed up in psychiatry outpatient clinics: one patient suffered from Generalized Anxiety Disorder and was treated with citalopram and another from Panic Disorder and was treated with psychotherapy. No patients had a possible pathological gambling, while 3 patients reported a substance use disorder (1 patient candidate to dupilumab and 2 patients in treatment with dupilumab for 1 year). No further statistical analyses were performed with regard to pathological gambling and substance use disorders in the light of no/few cases with these conditions.

**Table 1:** Clinical variables of the total sample and of the two groups identified by the treatment with dupilumab

Variables		Total sample (N = 66)	Candidates or treatment with dupilumab ≤ 1 month (N = 24)	Treatment with dupilumab for 1 year (N = 42)	p	q-FDR
Age		37.70 (±17.41)	35.67 (±18.30)	38.86 (±17.00)	0.478	0.637
Gender	Male	36 (54.5%)	13 (54.2%)	23 (54.8%)	0.963	0.963
	Female	30 (45.5%)	11 (45.8%)	19 (45.2%)		
Total IgE (kU/l)		1259.83 (±1875.03)	1370.88 (±1464.28)	1235.16 (±1971.57)	0.856	0.944
HADS-anxiety		5.02 (±4.45)	6.79 (±5.29)	4.00 (±3.58)	**0.027**	**0.041**
HADS-depression		4.82 (±4.53)	7.00 (±4.97)	3.57 (±3.78)	**0.002**	**0.003**
POEM		8.26 (±8.32)	14.87 (±9.72)	4.64 (±4.41)	**<0.001**	**0.002**
DLQI		5.91 (±7.32)	12.13 (±8.71)	2.36 (±2.61)	**<0.001**	**0.002**
SQ-NRS		1.45 (±2.72)	3.29 (±3.57)	0.40 (±1.33)	**0.001**	**0.002**
itch-INRS		3.91 (±3.27)	6.50 (±3.32)	2.43 (±2.14)	**<0.001**	**0.002**
PGA		1.55 (±1.26)	2.63 (±1.25)	0.93 (±0.75)	**<0.001**	**0.002**
EASI		9.30 (±12.43)	21.50 (±13.34)	2.33 (±2.85)	**<0.001**	**0.002**
IAT		32.45 (±10.53)	14.87 (±9.72)	4.64 (±4.41)	0.865	0.944

Means for continuous variables and frequencies for qualitative ones are reported in the table. Standard deviations for continuous variables and percentages for qualitative ones are reported in brackets. In bold statistically significant p and q-FDR. Q-FDR values from multiple comparison methods were based on Benjamin-Hochberg False Discovery Rate.

DLQI, Dermatology Life Quality Index; EASI, Eczema Area and Severity Index; FDR, false discovery rate; HADS, Hospital Anxiety and Depression Scale; IAT, Internet Addiction Test; IgE, immunoglobulin E; Itch-NRS, the Itch Numerical Rating Scale; PGA, Physician Global Assessment; POEM, Patient Oriented Eczema Measure; SQ-NRS, Sleep Quality Numeric Rating Scale.

Patients with clinically significant anxiety symptoms (compared to those without these symptoms) were found to have more severe AD (POEM: t = 3.20, *P* = 0.005; INRS: t = 2.36, *P* = 0.021; PGA: t = 3.31, *P* = 0.002; EASI: t = 2.54, *P* = 0.013), more sleep problems (t = 2.36, *P* = 0.028), worse quality of life (DLQI: t = 3.71, *P* = 0.001), more severe depressive symptoms (t = 5.35, *P* < 0.001), were more likely female (χ^2^ = 5.83, *P* = 0.016, OR: 4.13 [1.25–13.64]), and were less likely to have been treated with dupilumab for 1 year (χ^2^ = 4.99, *P* = 0.025, OR: 0.28 [0.09–0.88]).

Similarly, patients with clinically significant depressive symptoms (compared to those without depressive disorders) were found to have more severe AD (POEM: t = 3.81, *P* = 0.001; INRS: t = 3.54, *P* < 0.001; PGA: t = 3.77, *P* < 0.001; EASI: t = 3.36, *P* = 0.002), more sleep problems (t = 3.39, *P* = 0.003), worse quality of life (DLQI: t = 4.50, *P* < 0.001), more severe anxiety symptoms (t = 6.09, *P* < 0.001), were less likely to have been treated with dupilumab for 1 year (χ^2^ = 6.93, *P* = 0.008, OR: 0.23 [0.08–0.71]).

In contrast, no statistically significant differences were found in the severity of AD, sleep disturbance, depression, or quality of life for groups identified by the presence of significant IA symptoms (*P* > 0.05).

Patients, who had been in treatment with dupilumab for at least 1 year, showed less POEM (t = 4.79, *P* < 0.001), itch-INRS (t = 5.40, *P* < 0.001), PGA (t = 6.08, *P* < 0.001), EASI (t = 6.95, *P* < 0.001), DLQI (t = 5.36, *P* < 0.001), HADS-depression (t = 3.16, *P* = 0.002), HADS-anxiety (t = 2.30, *P* = 0.027), SQ-NRS (t = 3.82, *P* = 0.001) scores compared the counterpart. No statistically significant differences were identified in the two groups with regard to age (t = 0.71, *P* = 0.478), gender (χ^2^ = <0.01, *P* = 0.963), serum total IgE (t = 0.18, *P* = 0.856) and IAT scores (t = 0.17, *P* = 0.865) (Table 1).

## Discussion

This study had the objectives to evaluate the presence of psychiatric comorbidity in patients affected by AD and to evaluate the impact of a long-term treatment of dupilumab (1 year) on mental health.

Regarding the first point, a large number of our patients showed clinically significant symptoms of anxiety, depression and IA, while substance use disorders and pathological gambling would be less represented in our sample. As previously mentioned, in the last years an increasing literature has reported frequent comorbidity with affective disorders in patients with AD (Iannone *et al.*, 2022). In addition, our findings indicate that the severity of anxiety and depression is directly proportional to severity of skin disease, thus supporting the hypothesis that the frequent comorbidity between psychiatric and dermatological disorders can be the result of shared biological mechanisms (e.g. prominent inflammation), psychological impact of skin lesions and insomnia for nocturnal itch (Fabrazzo *et al.*, 2021; Ferrucci *et al.*, 2021). Furthermore, both AD and depression can be exacerbated by shared risk factors including vitamin D deficiency, stressful events, air pollution and overweight (Borroni *et al.*, 2022; Zhang *et al.*, 2022).

In contrast, this is the first article reporting an extremely frequent presence of IA in subjects affected by AD, while substance use disorders and pathological gambling would appear to be marginal. Authors of a recent article, reporting data from a German university hospital, found that 6.4% of patients with AD screened positive for drug use disorders, 4.5% for IA and 3.2% for pathological gambling (Pilz *et al.*, 2022). The discrepancy of results can be due to several factors including different tools to assess psychiatric conditions, cultural aspects and the fact that IA is not a clearly established diagnostic category (Pan *et al.*, 2020). In addition, IA seems to be independent from the severity of anxiety and depression as well as from the severity of dermatological disease. We did not investigate the reason of use of web by patients, but subjects affected by AD could search medical information on the internet. In this regard, a new entity has been recently proposed by some authors who defined it as ‘cyberchondria’, consisting in a compulsive tendency to search medical information on the web (Schenkel *et al.*, 2021). Cyberchondria seems to share clinical symptoms with illness anxiety disorder (Schenkel *et al.*, 2021), IA and obsessive-compulsive disorder (Starcevic *et al.*, 2020; Vismara *et al.*, 2020) and, as showed by our sample, less associated with severity of affective symptoms and dermatological disease. The patients who had a remission of both depressive/anxiety symptoms and AD could persist in seeking health care information on web for the worries to have relapses (Vismara *et al.*, 2020).

As expected patients who had received treatment with dupilumab for 1 year showed less severe AD as showed by less rating scale scores than the counterpart (Kamata and Tada, 2021). Furthermore, patients in treatment with dupilumab for 1 year reported less severe depressive and anxiety symptoms as well as a better quality of sleep than the other group. At this point two hypotheses could be formulated: the first is that the amelioration of dermatological symptoms and consequently of quality of life produce an improvement of psychiatric disorders; the second is that dupilumab has an effect also on affective symptoms, acting on shared underlying biological mechanisms. In support of this latter hypothesis, some authors reported the effectiveness of anti-inflammatory compounds for the treatment of depression alone or in comorbidity with autoimmune disease including dermatological conditions (Vasiliu, 2022). In addition, IL-4 was found to be correlated with severity of anxiety in subjects affected by AD (Gray *et al.*, 2020). On the other hand, Selective Serotonin Reuptake Inhibitors have been proposed by some authors as potential and promising compounds for the treatment of AD (Kiecka and Szczepanik, 2022).

Globally, the findings of this study indicate that an integrated approach including dermatologists and psychiatrists has the best benefit for patients affected by AD in the light of the high prevalence of psychiatric conditions in this population (Pompili *et al.*, 2021). Preventive strategies could focus on modifiable risk factors associated with an increased risk of both AD and affective disorders (e.g. diet, overweight or air pollution) (Iodice *et al.*, 2021). In addition, future research should confirm the potential effectiveness of anti-inflammatory drugs (e.g. dupilumab) or antidepressants (that have anti-inflammatory properties) on both AD and psychiatric conditions (Benedetti *et al.*, 2022).

The findings of the present article should be interpreted taking into account the following limitations:

1. The lack of randomization with an active comparator to assess the effect of dupilumab on psychiatric symptoms than other immune modulators;
2. Medical and psychiatric comorbidity (despite the latter was identified in only two patients) may have affected the mental health of the sample;
3. Previous pharmacotherapy may have influenced some rating scale scores (e.g. systemic corticosteroids and severity of mood symptoms);
4. The context of pandemic may have impacted on mental health of fragile subjects such as those affected by AD (Ingegnoli *et al.*, 2021).

Randomized controlled studies with larger multi-centric samples are needed to confirm the findings presented in this article.

## Acknowledgements

Conceptualization: SMF, ST, MB; Data Curation: GDT; Formal analysis: MB, GN; Investigation: LA, EB; Methodology: SMF, ST, MB; Project administration: MB; Software: MB; Supervision: MB, AVM; Validation: LA, EB; Writing – original draft: SMF, ST, GN, AC; Writing – review & editing: AC, MB. All authors have read and agreed to the published version of the article.

The study was conducted according to the guidelines of the Declaration of Helsinki and approved by the Institutional Review Board (or Ethics Committee) of Fondazione IRCCS Ca’ Granda Ospedale Maggiore Policlinico of Milan.

Informed consent was obtained from all subjects involved in the study.

The data presented in this study is available upon request to the corresponding author.

### Conflicts of interest

There are no conflicts of interest.
